# Geriatric Nutritional Risk Index Less Than 92 Is a Predictor for Late Postpancreatectomy Hemorrhage Following Pancreatoduodenectomy: A Retrospective Cohort Study

**DOI:** 10.3390/cancers12102779

**Published:** 2020-09-28

**Authors:** Naotake Funamizu, Kenji Omura, Yasutsugu Takada, Takahiro Ozaki, Kohei Mishima, Kazuharu Igarashi, Go Wakabayashi

**Affiliations:** 1 Department of Surgery, Ageo Central General Hospital, Saitama Prefecture, Ageo, Saitama 362-8588, Japan; omura@ach.or.jp (K.O.); x.10.r.baggio@hotmail.co.jp (T.O.); mishima0930@gmail.com (K.M.); kazuharu_igarashi_0604@yahoo.co.jp (K.I.); go324@mac.com (G.W.); 2 Department of HBP Surgery, Ehime University, Ehime Prefecture, Matsuyama 791-0295, Japan; takaday@m.ehime-u.ac.jp

**Keywords:** postpancreatectomy hemorrhage (PPH), geriatric nutritional risk index (GNRI), postoperative pancreatic fistula (POPF), pancreatoduodenectomy (PD)

## Abstract

**Simple Summary:**

The definite risk factor of postpancreatectomy hemorrhage (PPH) is still unknown in spite of a lethal complication of pancreatoduodenectomy (PD). In this study, we evaluated whether GNRI is a reliable marker for PPH following PD. The present study retrospectively evaluated 121 patients treated with PD at Ageo Central General Hospital in Japan. Ten patients had developed PPH. Among them, the patients were divided into bleeding group (*n* = 10) and non-bleeding group (*n* = 111). The bleeding group had significantly low geriatric nutritional risk index (GNRI) values compared to those in the non-bleeding group (*p* = 0.001). The cut-off value of GNRI was determined by 92 accounting for a sensitivity 80.0%, specificity 82.9% using receiver operating characteristic curve analysis. A GNRI of <92 was statistically identified as an independently risk factor of PPH risk following PD.

**Abstract:**

Postpancreatectomy hemorrhage (PPH) is the most lethal complication of pancreatoduodenectomy (PD). The main risk factor for PPH is the development of a postoperative pancreatic fistula (POPF). Recent evidence shows that the geriatric nutritional risk index (GNRI) may be predictive indicator for POPF. In this study, we aimed to evaluate whether GNRI is a reliable predictive marker for PPH following PD. The present study retrospectively evaluated 121 patients treated with PD at Ageo Central General Hospital in Japan between January 2015 and March 2020. We investigated the potential of age, gender, body mass index, serum albumin, American Society of Anesthesiologists classification (ASA), diabetes mellitus and smoking status, time taken for the operation, estimated blood loss, and postoperative complications (POPF, bile leak, and surgical site infections) to predict the risk of PPH following PD using univariate and multivariate analyses. Ten patients had developed PPH with an incidence of 8.3%. Among them, the patients were divided into bleeding group (*n* = 10) and non-bleeding group (*n* = 111). The bleeding group had significantly lower GNRI values than those in the non-bleeding group (*p* = 0.001). We determined that the cut-off value of GNRI was 92 accounting for a sensitivity 80.0%, specificity 82.9%, and likelihood ratio of 4.6 using receiver operating characteristic curve analysis. A GNRI of <92 was statistically associated with PPH in both univariate (*p* < 0.001) and multivariate analysis (*p* = 0.01). Therefore, we could identify that a GNRI < 92 was an independently potential predictor of PPH risk following PD. We should alert surgeons if patients have low level GNRI before PD.

## 1. Introduction

Pancreatoduodenectomy (PD) for malignant hepatobiliary pancreatic tumors is the standard of care. However, the perioperative mortality rate of 5% has remained unchanged over the last few decades despite improved surgical techniques and advances in surgical devices [1]. Furthermore, it has been suggested that the morbidity rates range from 30% to 60% [2]. Among them, postpancreatectomy hemorrhage (PPH) is a rare, but potentially fatal complication following PD. The incidence of PPH is reported to occur between 3–16% with a mortality rate ranging from 16–38% [3,4,5]. Recent advancements in endovascular interventions, including covered stents and embolization techniques, have contributed to reduced mortality rate. The International Study Group for Pancreatic Surgery (ISGPS) defined of early and late PPH based on bleeding onset (early if <24 h after surgery, delayed when >24 h). In this study, we took particular note of late PPH which is occurring >24 h after PD. The reported etiology of late PPH is mainly associated with vessel erosion caused by postoperative pancreatic fistula (POPF), bile leakage, intra-abdominal infection with vessel involvement, or intraoperative vascular injury and pseudoaneurysm formation in the postoperative course [6]. PPH due to pseudoaneurysm development was associated in up to 30% of cases [7], and most commonly involves the gastroduodenal artery. It is felt that vascular damage elicited by surgery leads to increased pressure resistance causing pseudoaneurysm development, increasing the risk for PPH. Among all causes of PPH, the majority are caused by POPF accounting for up to 70% of all cases [8]. Additionally, a recent study showed that POPF was involved in 80% of PPH cases [9]. Due to this high morbidity and mortality associated with PPH, it is essential for surgeons to be able to accurately predict the risk and occurrence of PPH. Recently, the geriatric nutritional risk index (GNRI) was developed as a nutritional screening tool with easily accessible and non-invasive assessments [10]. The GNRI only requires body weight, height, and serum albumin levels. Previously, we showed that the GNRI is associated with increased risk of surgical site infections and POPF [11]. As a result, we hypothesized that GNRI could be useful to assess the risk of PPH in those following PD. There have been several studies describing late PPH, but there has been no focus on prevention, prediction, early diagnosis, treatment approaches, and prognosis. Thus, the aim of study was to determine the association of GNRI and its predictive nature of PPH following PD, which may improve early recognition, treatment, and outcomes. 

## 2. Results

### 2.1. Patient Characteristics and Univariate Analysis for PPH risk Following PD 

Between January 2015 and March 2020, 121 patients underwent PD at Ageo Central General Hospital (Saitama, Japan). Of these patients, 103 and 18 patients had undergone open PD or pure robotic PD respectively. The indications for PD included adenocarcinoma of the pancreas or periampullary region in 100 patients (82.6%), the remaining cases involved cystic pancreatic lesions in 14 patients (11.6%), neuroendocrine tumors in 1 (duodenum: 0.8%) and 2 patients (pancreas: 1.7%), chronic pancreatitis in 1 patient (0.8%), periampullary adenoma in 1 patients (0.8%), metastatic chondrosarcoma in 1 patient (0.8%), and duodenal gastrointestinal stromal tumor in 1 patient (0.8%). Postoperatively, 10 patients (8.3%) underwent interventional radiography guided procedures leading to late PPH. Table 1 summarizes the univariate analysis with clinical variables of patients in the bleeding group (*n* = 10, 8.3%) and non-bleeding group (*n* = 111, 91.7%). Average time to bleeding onset following PD was 20.1 days (range, 6–58 days) after PD. Three patients had grade C, and 7 patients had grade B bleeding. Sentinel bleeding was identified in 5 patients (50.0%). Arteries associated with bleeding were as follows: common hepatic artery in 4 patients; gastroduodenal artery stump in 3 patients; right hepatic artery in 2 patients; middle colon artery in 1 patient respectively. All patients were successfully treated either by covered stent placement or by artery embolization. Fortunately, no deaths were attributed directly to PPH, but 3 patients died because of aspiration pneumonia after PPH was identified and corrected. There were no statistically significant differences in age, BMI, ASA classification, presence of periampullary adenocarcinoma, ongoing tobacco use, alcohol intake, presence of diabetes mellitus, neoadjuvant chemotherapy, preoperative biliary drainage, approach used for PD, time taken for the operation, estimated blood loss, need for blood transfusion, portal vein resection, and bile leakage between bleeding and non-bleeding groups. On the other hand, statistically significant differences were observed for gender (*p* = 0.01), preoperative albumin level (*p* < 0.001), GNRI (*p* = 0.001), POPF (*p* = 0.005), intraabdominal infection (*p* = 0.02), and duration of postoperative hospital stays (*p* < 0.001) in Table 1. 

### 2.2. Receiver Operating Characteristic (ROC) Curve Analysis 

**Figure 1:**
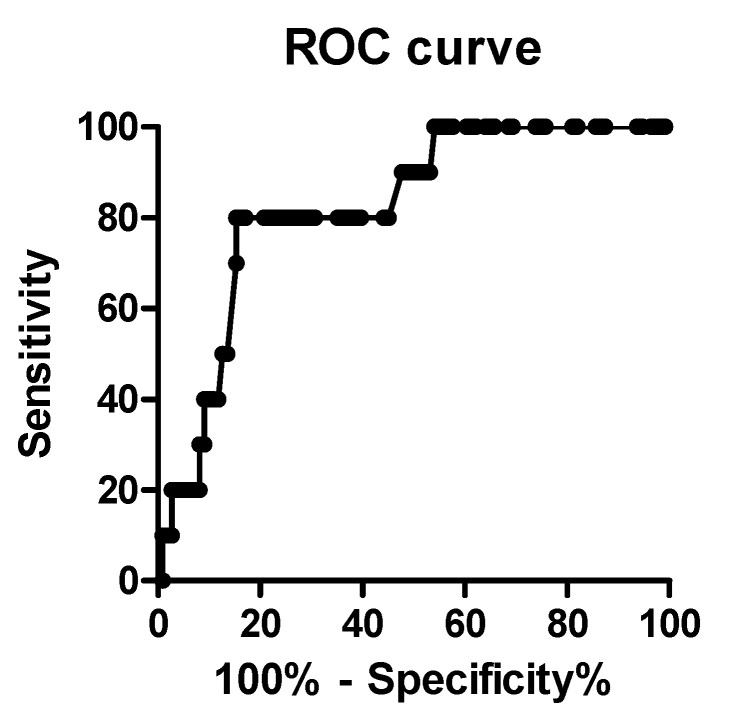
ROC curve analysis showed that GNRI was selected by 92 as an optimal cut-off value with a sensitivity 80.0% and a specificity 82.8%.

To choose the optimal GNRI cut-off value for the prediction of PPH, ROC curve analysis was performed. With an area under the curve (AUC) of 0.82 (95% confidence interval: 0.71–0.94), the cut-off value of GNRI of 92 was identified. This value had a sensitivity of 80.0% and a specificity of 82.8%. Secondary, patients were divided into two groups: group A (GNRI ≥ 92, *n* = 94) and group B (GNRI < 92, *n* = 27) according to the determined cut-off value of 92 (Figure 1). 

### 2.3. Univariate Analysis of a GNRI < 92 for PPH 

Univariate analysis showed that a GNRI value < 92 was potentially marker of PPH risk following PD. The developed PPH rate was 2.1% in group A and 29.6% in group B. The incidence of PPH was significantly higher in group B than in group A (*p* < 0.001).

### 2.4. Multivariable with Logistic Regression Analyses

Logistic regression analysis showed that a GNRI value < 92 was an independent predictive factor for PPH (Table 2).

## 3. Discussion 

PD is relatively cumbersome and a highly invasive operation for patients among gastroenterological procedures with a reportedly morbidity ranging from 40–60%, despite advances in surgical skill and postoperative management [12,13,14]. The most frequent postoperative complications are developments of POPF, delayed gastric empty, intraabdominal infection, and PPH. Especially, PPH is the most urgent and potentially life-threatening complication after PD [12]. PPH was defined and classified by the International Study Group of Pancreatic Surgery (ISGPS). PPH occurring within 24 h of the pancreatectomy was defined as early PPH which was usually caused by accidental technical failure during the operation. On the other hand, PPH occurring after 24 h was defined as late PPH, which might be associated with postoperative complications including pseudoaneurysms, ulcers, and erosive vessels caused by POPF [15]. In this study, we had 10 patients (8.3%) who developed PPH following PD, which is slightly higher than previous reports and likely reflects the older age of our patients compared to those studies [16]. The development of PPH has also been associated with poor overall survival likely due to delays in chemotherapy for those with malignancies [17]. Therefore, the primary goal of all surgeons is to prevent these postoperative complications, and when present, intervene early. Early detection of PPH is primarily dependent upon the sentinel bleeding as an initial sign of PPH, which is usually seen by decreasing hemoglobin or increase abdominal pain. In fact, sentinel bleeding precedes severe PPH in more than 50% of patients [18]. In this study, 50% of patients had early signs of bleeding leading to early PPH detection. Arteriography with embolization is the most commonly used treatment approach, but recently, covered stents have been used in lieu of embolization without vessel obstruction. Interventional radiology is a less invasive approach compared to surgical procedure. Previous meta-analysis has shown that surgical interventions for PPH are associated with higher rates of morbidity and mortality compared improved success with interventional radiologic approaches [5]. The success rate of endovascular stenting for PPH is reported between 82–100%, but should be selected carefully due to high rates of re-bleeding approaching 7–30% [19]. In this study, all patients who developed PPH were treated by interventional radiology endovascular with covered stent replacement and all survived.

Recent reports have shown that potential risk factors for PPH following PD include body mass index (BMI), intraoperative transfusion, portal vein resection, POPF, preoperative biliary drainage, absence of diabetes, and lower serum albumin level [5,20,21]. However, the definite risk factor for PPH following PD was still unknown. Among them, we focused on GNRI, which includes albumin and BMI, primarily because of availability of the required parameters and lack of invasiveness. Until now, it had been reported that lower GNRI was associated with increased risk of surgical site infections in patients following PD [11], and POPF in patients with distal pancreatectomy at a different hospital [22]. In detail, a lower GNRI value was strongly associated with a higher risk of surgical site infections, supporting the use of nutritional assessment prior to an elective surgical procedure. In addition, GNRI is also a potential predictor for the development of POPFs. The GNRI was originally developed to predict prognosis through nutritional status for hospitalized elderly patients as an objective nutritional screening tool [8]. Recent evidence showed that particularly those patients with severe chronic renal failure and chronic heart failure had poorer outcomes [23,24]. More recently, several studies have shown that other nutritional assessments in those with pancreatic carcinoma, particularly the presence of sarcopenia, have been published and are independent predictors of poor outcomes [25]. Subsequently, the prognostic nutritional index (PNI) which is calculated based on the serum albumin value and peripheral blood lymphocyte count and is an indicator of nutritional and immune function status, was found to be a prognostic marker in localized pancreatic carcinoma after chemotherapy [26]. On the other hand, several reports showed the association between GNRI and postoperative complications. For instance, Wang et al. showed that GNRI could be used to risk stratify elderly patients who underwent radiotherapy or definitive concurrent chemoradiotherapy [27]. In addition, our previous report revealed that GNRI has been identified as a risk factor for SSIs in patients after PD [11]. Moreover, Kushiyama et al. showed that GNRI is useful marker in predicting postoperative complications for elderly patients after gastrectomy [28]. Additionally, recent reports revealed that GNRI is associated with prognosis for cancer patients. Iguchi et al. reported that GNRI is helpful as an independent prognostic factor for patients with colorectal liver metastasis [29]. Hirahara et al. also showed that GNRI is significantly associated with overall survival and cancer specific survival in elderly gastric cancer patients, and GNRI is an independent predictor of overall survival [30]. Therefore, in this study, we aimed to identify whether GNRI could become a predictive marker for the risk of PPH following PD. As a next step, we will investigate whether GNRI can predict the prognosis for the patient after PD. The present study showed that a lower GNRI value (<92) strongly correlates with a higher risk of PPH. This correlation will support the feasibility of nutritional assessment using body weight, height and albumin value preoperatively. Thus, surgeon should pay particular attention to their patients with low GNRI scores following PD, and if sentinel bleeding is identified, ruling out PPH is essential. 

Our current study has several limitations that must be considered. Firstly, our sample size is relatively small and accounts for the lack of statistical power for our analyses. Secondly, this study is a retrospective cohort, which is potentially fraught with biases. Thirdly, GNRI has not been compared with other nutritional assessment tools, and a more comprehensive prospective randomized control trial could be helpful to determine the utility of GNRI has a risk factor for PPH following PD. 

Finally, although GNRI was initially developed as a nutritional screening tool to assess the nutrition-related prognosis in elderly hospitalized patients, it has been reported to predict mortality in elderly patients with chronic pulmonary disease, chronic renal failure, and chronic heart failure. In this time, we hypothesize that GNRI might be a promising predictor of PPH in those who underwent PD which can be easily obtained from simply laboratory data and physical measurements without an invasive and specific approach.

## 4. Materials and Methods

### 4.1. Patients 

Between January 2015 and March 2020, 121 patients underwent open PD for hepatobiliary pancreatic or duodenal tumors in Ageo Central General Hospital. Exclusion criteria were exploratory laparotomy or choledochojejunostomy. All surgical procedures were performed by surgeons with substantial experience in pancreatic surgery. The definition of PPH and POPF were defined and clarified according to the International Study Group for Pancreatic Surgery (ISGPS) in 2016 [15]. In this study, grade B and grade C are defined as POPF. Clinical data were collected from both inpatient and outpatient medical records. This study was approved by the Ethical Committee of Ageo Central General Hospital in 2019 (Approval number: AMG790) and followed the Declaration of Helsinki as revised in 2013. All participants including retrospectively registered patients or their guardians, provided verbal consented for the use of their medical information for scientific research. 

### 4.2. Collection of Clinical and Laboratory Data

As preoperative parameters, clinical data was obtained and analyzed, which included demographic variables (gender and age), anthropometric parameters (height, weight, and body mass index (BMI)), American society of anesthesiologist (ASA)’s physical status classification, smoking history, alcohol intake, comorbidities, location of the tumors, neoadjuvant chemotherapy, preoperative biliary drainage, preoperative albumin value, GNRI. Moreover, intra- or postoperative parameters, portal vein resection and reconstruction, time taken for the operation, estimated blood loss, need for blood transfusion, POPF, intraabdominal infection, and PPH were collected from individual medical records. 

### 4.3. Definition of GNRI

GNRI was measured using body weight, height and serum albumin. Those data were obtained on admission as follows: *GNRI = [14.89 × serum albumin (g/L)] + [41.7 × present/ideal body weight (kg)])*. Ideal body weight was defined as 22 *×* patient height (m)^2^. If present body weight was higher than the ideal body weight, present/ideal body weight was set as 1.

### 4.4. Perioperative Management and Surgical Technique for PD

All patients preoperatively underwent routine blood tests including serum albumin assessment, and a physical examination including body weight and height. Coagulation status was assessed preoperatively. Prophylactic antibiotics were administered through a peripheral vein before anesthetic induction. All patients underwent PD in the supine position under general anesthesia. Proton pump inhibitors and protease inhibitors were administered to all patients immediately after PD. Amylase values from ascitic fluid obtained from drainage tube was measured every other day until removal of the drains. In addition, dynamic CT was performed within four or seven days postoperatively to assess the presence of fluid collections before the drainage tubes were removed. PD pattern depended on the tumor location and surgeon preference. Portal vein resection and reconstruction were performed if the tumor could not be dissected from the superior mesenteric vein or the portal vein. Lymphadenectomy was performed in all patients routinely. The ligamentum teres hepatis was used to cover the anastomotic site of pancreatojejunostomy surface. The gastroduodenal artery stump was routinely ligated with nonabsorbable 3–0 suture ligation and additionally ligated with 3–0 thread. Reconstruction of PD was performed using Child’s method. All patients underwent pancreatojejunostomy anastomosis to the exclusion of pancreatogastrostomy. 

### 4.5. Statistical Analysis 

All statistical significances were calculated by Graphpad Prism v5.0 (Graphpad Software Inc., La Jolla, CA, USA) and SPSS (SPSS Inc., Chicago, IL, USA). Statistical analyses were performed using the Fisher’s exact test or Chi-squared test. A receiver operating characteristic (ROC) curve was calculated to identify the optimal cut-off value of GNRI for evaluating the risk of PPH. Potential risk factors for PPH were evaluated using univariate and multivariate analyses. Independent risk factors for PPH were identified by a logistic regression. Odds ratios with 95% confidence intervals were also calculated. The probability of *p* < 0.05 was considered to be statistically significant.

## 5. Conclusions

A lower GNRI value might be a potential risk factor of PPH in patient following PD. We believe that GNRI—calculated using body weight, height, and albumin level—is a valuable, non-invasive, and inexpensive tool that should be used to evaluate the risk of PPH. 

## Figures and Tables

**Table 1:** Results of univariate analysis. The analysis revealed significantly higher incidence of PPH in those with GNRI < 92.

Characteristics	PPH Group	Non-PPH Group	*p*-Value
	(*n* = 10)	(*n* = 111)	
Gender			
Male *vs* Female	10 vs. 0	69 vs. 42	*p* = 0.01
Age (years)	76.1 ± 2.0	70.8 ± 1.1	*p* = 0.15
Body mass index	22.7 ± 0.7	22.1 ± 0.3	*p* = 0.55
ASA classification			
1 or 2	8 (80.0%)	97 (87.4%)	*p* = 0.51
3	2 (20.0%)	14 (12.6%)	
Pancreatic or periampullary	8 (80.0%)	91 (82.0%)	*p* = 0.88
adenocarcinoma (%)			
Smoking habit (%)	2 (20.0%)	8 (7.2%)	*p* = 0.19
Alcohol intake (%)	0 (0.0%)	3 (2.7%)	*p* = 0.60
Diabetes mellitus (%)	1 (10.0%)	36 (32.4%)	*p* = 0.14
Neoadjuvant chemotherapy (%)	0 (0.0%)	9 (8.1%)	*p* = 0.35
Preoperative biliary drainage (%)	6 (60.0%)	35 (31.5%)	*p* = 0.09
Preoperative albumin (g/L)	3.3 ± 0.1	3.9 ± 0.1	*p* < 0.001
Robotic/open PD	2/8	16/95	*p* = 0.63
GNRI	89.2 ± 1.9	98.3 ± 0.8	*p* = 0.001
92<	8	19	*p* < 0.001
92≥	2	92	
Time taken for the operation (min)	578.1 ± 42.2	497.2 ± 15.1	*p* = 0.13
Estimated blood loss (ml)	712.7 ± 103.5	656.2 ± 63.3	*p* = 0.69
Blood transfusion (%)	2 (20.0%)	25 (22.5%)	*p* = 0.85
Portal vein resection (%)	0 (0.0%)	6 (5.4%)	*p* = 0.45
Bile leakage (%)	2 (20.0%)	5 (4.5%)	*p* = 0.09
Postoperative pancreatic fistula (%)	7 (70.0%)	27 (24.3%)	*p* = 0.005
Grade B or C (%)			
Rate of intraabdominal infection (%)	6 (60.0%)	24 (22.5%)	*p* = 0.02
Postoperative hospital stays (day)	49.0 ± 8.2	22.9 ± 5.5	*p* < 0.001

**Table 2:** Multivariate analysis by logistical regression. GNRI < 92 was an independent risk factor for PPH following PD.

Variables	Odds Ratio	95% Confidence Interval	*p*-Value
Gender	0.001	Not available	*p* = 0.97
Intra-abdominal abscess	0.58	0.01–0.46	*p* = 0.65
POPF	4.11	0.44–38.29	*p* = 0.21
GNRI < 92	0.07	0.01–0.46	*p* = 0.006
